# Acceptability and Feasibility of Best Practice School Lunches by Elementary School-Aged Children in a Serve Setting: A Randomized Crossover Trial

**DOI:** 10.3390/ijerph17176299

**Published:** 2020-08-29

**Authors:** Jillian M. Joyce, Kyleen Harris, Emily L. Mailey, Richard R. Rosenkranz, Sara K. Rosenkranz

**Affiliations:** 1 Department of Nutritional Sciences, Oklahoma State University, Stillwater, OK 74078, USA; 2 Department of Food, Nutrition, Dietetics and Health, Kansas State University, Manhattan, KS 66506, USA; kyleenk@ksu.edu; 3 Department of Kinesiology, Kansas State University, Manhattan, KS 66506, USA; emailey@ksu.edu; 4 Department of Food, Nutrition, Dietetics and Health, Physical Activity and Nutrition Clinical Research Consortium, Kansas State University, Manhattan, KS 66506, USA; ricardo@ksu.edu (R.R.R.); sararose@ksu.edu (S.K.R.)

**Keywords:** National School Lunch Program, dietary quality, child nutrition

## Abstract

Background: National School Lunch Program (NSLP) standards have improved school lunch dietary quality (DQ), however, further improvements could be made. Acceptability and feasibility of higher DQ are potential barriers. Thus, the purpose is to compare acceptability and feasibility of best practice (BPSL, optimizing DQ) with typical school lunches (TSL, meeting minimum NSLP standards) served separately and concurrently. Methods: Forty elementary school-aged participants were recruited for a randomized crossover trial. Participants attended three meal conditions (MC) choosing one of two meal types—MC1) BPSL1/BPSL2, MC2) TSL1/TSL2, MC3) BPSL/TSL. Acceptability included taste test surveys, weighted plate waste assessments, and hunger scales. Feasibility included meal cost, time, and skill and equipment requirements. Results: There were no significant differences in total taste test score, average total plate waste, or change in hunger (*ps* > 0.017) before or after adjusting for covariates. TSL was selected significantly more often in MC3 (TSL = 83.3%, BPSL = 16.7%, *p* = 0.001). Meal cost (*p* = 0.783) and skill and equipment requirements were not significantly different between meal types. BPSL required significantly longer preparation time (TSL = 60 ± 25 min, BPSL = 267 ± 101 min, *p* = 0.026). Conclusions: Results indicate few differences in acceptability and feasibility between BPSL and TSL. This study could inform decision and policy-makers seeking to improve school lunch DQ and acceptance of higher DQ meals.

## 1. Introduction

The Healthy, Hunger-Free Kids Act led to substantive changes to the National School Lunch Program (NSLP) in January 2012 that improved the dietary quality (DQ) of school lunches [1]. These changes required schools participating in the NSLP to provide more fruits and vegetables, vary vegetable color and type, increase provision of whole grains, decrease added sugar and sodium, and lower saturated fat content [2,3]. These changes represent a shift in the focus of the NSLP nutrition standards toward food groups and DQ, and away from individual nutrients. The shifting of focus toward DQ is supported by recent changes in other government nutrition entities. The Healthy Eating Index (HEI), created by the USDA Center for Nutrition Policy and Promotion (CNPP) and the National Cancer Institute of the National Institutes of Health, focuses mainly on food components, similar to the NSLP changes, for evaluating DQ, not individual nutrient content [4]. The 2015 Dietary Guidelines for Americans healthy meal pattern recommendations, and the 2016 Child and Adult Care Food Program best practices, also support focusing on food groups and DQ, as compared to individual nutrients [5,6].

The aforementioned changes in the NSLP are also supported by previous research, as many studies have shown the benefits of improved DQ through changes in dietary patterns and school lunches. The health benefits of improving DQ in childhood include a lower risk of overweight, obesity, and numerous chronic diseases, in childhood and adulthood [7,8,9]. The academic benefits of higher DQ school lunches include improved on-task time and increased alertness during the school day [10], as well as higher scores in reading fluency and comprehension [11], decreased authorized absenteeism [12], and optimized child cognitive and behavioral function [13].

Implementation and adoption of these new standards has been slow, and there are now also efforts to reverse DQ advances made in the new standards (i.e., higher target sodium levels, allowing low-fat flavored milk, and providing less whole grain-rich products) [14]. The School Nutrition Dietary Assessment IV (SNDAIV) is a report that evaluates the nutrient content of average school meals and competitive foods using a representative sample of US schools, comparing them to school meal standards and selected aspects of the Dietary Guidelines for Americans. The most recent report showed that implementation of the NSLP updates was poor, with only 14% of schools in compliance at the end of the first year [15]. Slow implementation, and now relaxation of the improved standards, is reportedly due to barriers to implementing higher DQ school lunches [14,15].

Several studies have examined the perceived barriers to implementation of higher DQ school lunches from the perspective of principals, school foodservice directors, and school foodservice personnel [16,17,18]. A large observational study by Nollen et al. (2007) investigated the perceptions of high school personnel regarding the relationship between the school food environment and obesity [16]. Related to feasibility, the study found that school personnel felt that they were doing the best they could with available resources, that barriers to offering healthier food items included cost and waste, and that wellness plans would be better implemented if personnel were given the proper resources, including money. Related to acceptability, school personnel felt a need to maintain high participation rates, were concerned about waste, and wanted to be liked and appreciated [16]. Another cross-sectional survey by Brouse et al. (2009) used a random sample of 259 school foodservice directors and investigated perceptions of barriers to improving the nutrition status of schoolchildren [17]. Perceived barriers to serving healthier food items included lack of time, the high cost of fruits and vegetables, pressure to serve foods that schoolchildren liked as compared to healthful foods, and financial concerns regarding healthy food offerings [17]. A final cross-sectional study by Fulkerson et al. (2002) surveyed 235 urban school foodservice personnel in Michigan regarding perceptions of interactions with students, barriers to suggesting healthful foods to students, and perceptions of student nutrition [18]. Perceived barriers to suggesting healthful food items included lack of time and students having made their decisions regarding less healthful items. Additionally, school foodservice personnel felt that reasons for students not choosing healthful food items included general dislike of those types of foods [18]. A recurring theme was concern over lower acceptability and feasibility of school lunch menus offering more healthful food items. These barriers were reported as perceived, but the extent to which these perceived barriers are real is uncertain.

There has been some previous research that has investigated the existence of these barriers following changes to school foodservice as a result of implementation of the updated NSLP nutrition standards, and thus higher DQ school lunches. Following the January 2012 update and subsequent major school lunch content changes, several studies evaluated plate waste differences [19,20,21,22,23,24], few studies investigated cost differences [25,26,27], and no known studies determined taste differences or additional feasibility differences (i.e., staffing, preparation time, and equipment needs) pre- and post-implementation. Additionally, current data may not be accurate regarding acceptability and feasibility, as only 14% of schools in 2013 were fully compliant with updated NSLP guidelines, when most of these studies occurred [15]. Further, studies of children thus far have neglected to look at true preferences for less healthful, lower DQ foods, as compared to healthier, higher DQ food options. Thus, there is a critical gap in current research on the acceptability and feasibility of providing higher DQ school lunches.

The purpose of the current study was to fill this critical gap in research knowledge by assessing the acceptability and feasibility of lunches that are high in DQ. The aims were to determine (1) whether there were differences in the acceptability of best practice school lunches, as compared with typical school lunches; (2) whether there were differences in the feasibility of best practice school lunches, as compared with typical school lunches; and (3) whether the presence of both meal types in one meal setting (choice) influenced the acceptability of the best practice school lunches. With these questions answered, this study could provide important information to decision- and policy-makers with regard to the need for, and practicality of, providing higher DQ school lunches.

## 2. Materials and Methods

### 2.1. Participants

The population of interest for this randomized crossover trial was elementary school-aged children in grades kindergarten through fifth (K–5). Participants were recruited from four local school districts, consisting of approximately 8300 eligible participants, using informational flyers that were emailed to parents and guardians via school wellness committees and posted on Facebook for public sharing. Information was also disseminated to Kansas State University faculty and staff via internal communication. Interested guardians contacted the principal investigator to express interest, and participants were screened via email for inclusion and exclusion criteria. Inclusion criteria included attendance at a school receiving NSLP reimbursement, being in grades K‒5, and parent/guardian willingness to transport the participant to all meal sessions. Exclusion criteria included having food allergies, currently receiving nutrition therapy, being home schooled or attending a school not participating in the NSLP, and not being available to participate in all three meal sessions. Eligible children were randomly assigned to one of three groups by random number generator, received a tray ID number (i.e., participant ID), and invited to come to Kansas State University for full screening and baseline assessment. Participants completing the study received a $25 gift card for a local grocery store, a printed cookbook with copies of study recipes, and a certificate of participation. IRB approval was obtained from Kansas State University Committee on Research Involving Human Subjects (proposal #8938). Informed consent and assent were obtained from guardians and participating children.

### 2.2. Sample Size and Power Calculations

With type 1 error rate set at 0.05 and power at 0.8, a sample size of four participants per group (i.e., 12 total participants) was needed for adequate power, based on plate waste differences from a study by Marlette et al. (2005) that evaluated school lunch plate waste differences between students who did and did not purchase competitive foods [20]. The current study aimed for 40 participants with anticipation of a 20–25% dropout rate and also allowing for adequate power to conduct multiple-comparisons across several dependent variables.

### 2.3. Study Design

**Figure 1:**
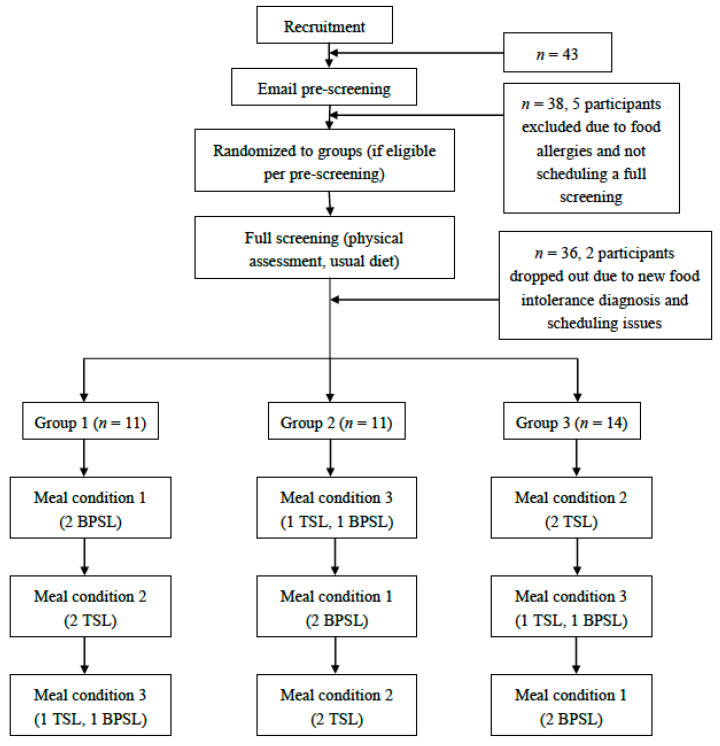
Flow chart of randomized crossover study design (typical school lunch, TSL; best practice school lunch, BPSL).

This study was a randomized crossover trial, where participants were randomized to one of three groups. Each group was assigned to receive three meal conditions comprising different meal types in a different and specific order, to control for an order or carryover effect. Meal conditions were provided, such that each group attended one session every three weeks. A flow chart of the overall study design can be found in Figure 1. Randomized participants attended a physical assessment and full screening before beginning any meal conditions.

Each meal condition consisted of a particular meal type based on DQ with two levels, (1) typical school lunch (TSL) and (2) best practice school lunch (BPSL). The TSL consisted of meals similar to those found in typical school lunches that were accessed from menus posted on local school foodservice websites (i.e., chicken tenders, hamburger, pizza, etc.). Each TSL met minimum NSLP nutrition standards, with average DQ (HEI score of 70–75/100). The BPSL consisted of meals that incorporated Child and Adult Care Food Program best practices [13] and 2015 Dietary Guidelines for Americans healthy meal pattern recommendations [12] and sought to maximize HEI 2010 scoring components [11]. Each BPSL had optimal DQ (HEI score of 90–95/100). All meals were created equally, aside from DQ, meeting all NSLP nutrition standards for the K–5th grade age group. Meal conditions were created by one of the researchers (JJ), a Registered Dietitian with expertise in creating best practice childcare and school lunch menus and with expertise in typical school lunch DQ from analyzing large samples of school lunch menus. At each meal session, acceptability (taste test survey, plate waste assessment, change in hunger) and feasibility (meal cost, preparation time, skill and equipment requirements) of the meals were determined.

Levels of the meal type were utilized to create three different lunch conditions. Meal condition one (MC1) consisted of a choice between two BPSL options. Meal condition two (MC2) consisted of a choice between one BPSL option and one TSL option. Meal condition three (MC3) consisted of two TSL options. The meals served for each meal condition can be found in Table 1.

**Figure 2:**
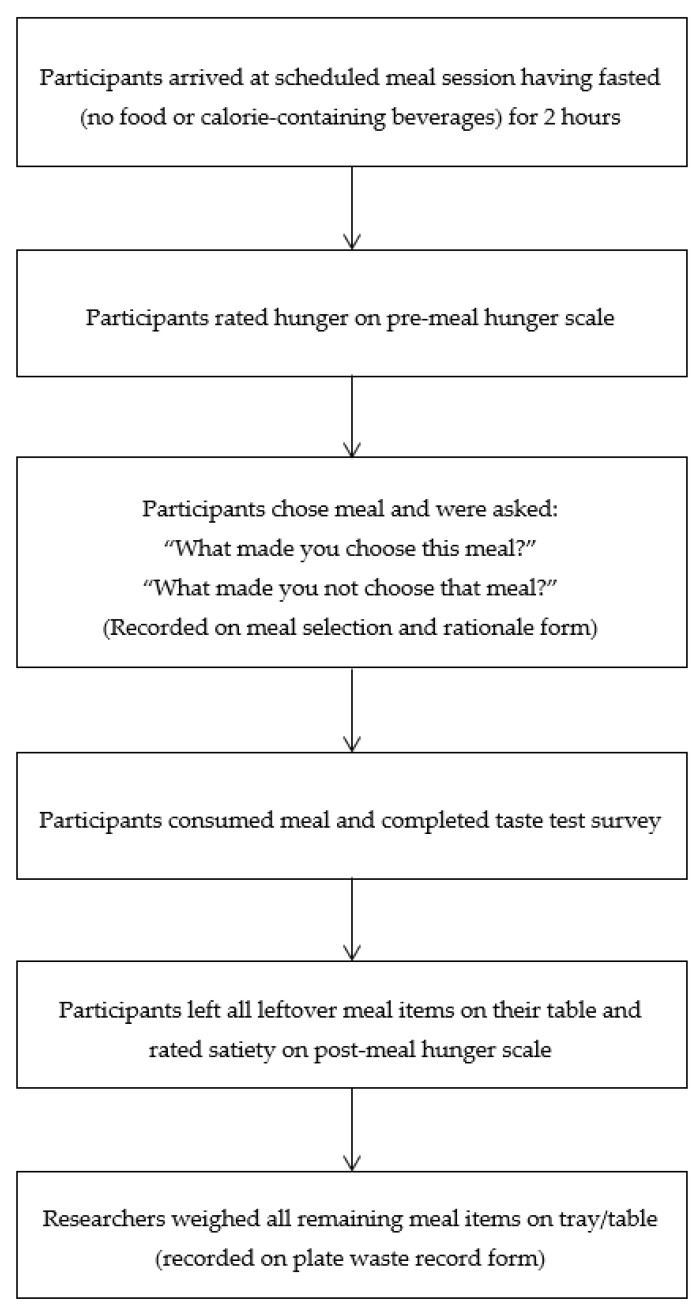
Meal session flow chart.

Following completion of the nine, scheduled meal sessions, make-up sessions were offered in order by meal condition (i.e., meal condition one first, meal condition two second, meal condition three third). Each meal session followed the same general procedure, which was designed to be similar to a typical school cafeteria, and lasted approximately 20–30 min. A flow chart of the meal sessions can be found in Figure 2.

### 2.4. Data Collection

At the pre-screening and initial assessment appointment, informed consent, both written guardian consent and written and oral child assent were obtained. Height, weight, and waist circumference measurements were obtained by two trained researchers. Detailed protocols for obtaining these measurements can be found in an article by Guagliano and Rosenkranz (2012) [28]. Two measurements were taken for each anthropometric characteristic, and the average value of the two measurements was used for analysis. Body mass index (BMI) percentile was determined using the Centers for Disease Control and Prevention (CDC) BMI percentile calculator for children and teens [29]. Usual diet was determined via 24 h dietary recall using the Automated Self-Administered 24 h (ASA24) Dietary Assessment Tool by the National Cancer Institute (version 2016, US Department of Health and Human Services, Washington, DC, USA) [30]. Guardians completed the dietary recall with participant assistance. Basic medical history was obtained from the guardian consisting of information about any known drug nutrient interactions, food allergies, issues with chewing or swallowing, nutrition therapy utilization, and conditions influencing diet but not receiving nutrition therapy. This information was obtained to ensure the participant was safe to consume all meals and to ensure that the participant was not limited in meal selection.

#### 2.4.1. Acceptability

Meal selection was assessed at meal condition 3, which included one BPSL and one TSL. As participants moved through the service line, their meal choice was recorded, and they were also asked why they chose what they did, and why they did not choose the other meal option. The meal selected, and selection rationale, were recorded along with tray ID number.

Taste test evaluation was performed at each of the three meal sessions using a modified version of the USDA, Food and Nutrition Services, Child Nutrition Programs, Team Nutrition try-day taste-testing ballot [31,32]. An example of the survey can be found in Figure S1. The form was provided with each tray and coded to match the tray ID number. Participants were asked to complete the form either during or after the meal, but before leaving the testing area. Smiley faces represented a 5-point Likert scale for responses to each question. These were coded for analysis (i.e., full frown/really dislike = 1, half frown/somewhat dislike = 2, flat face/neutral feelings = 3, half smile/somewhat like = 4, full smile/really like = 5). Scores for appearance, smell, taste, and desire to serve at school were recorded individually and also totaled to create a total taste test score. Researchers were present in the room during meal sessions to ensure that no food was discarded, and that all forms were completed and remained with the trays. Researchers were trained to not ask research related questions or interact with participants in such a way as to encourage or favor a particular meal option. Participants went through the service line individually and sat at individual tables facing the front of the eating area, as to minimize the impact of social pressures on acceptability measures.

Plate waste assessment was determined at each meal session using a modified method from several prior research studies investigating plate waste in school and adult care food program settings [23,33,34,35] and validated by the Rutgers Department of Nutritional Science and Extension Specialists [35]. Trays were numbered by trained researchers with the participant’s unique tray ID number. Trashcans were removed from the serving area, to ensure all plate waste remained on trays for assessment. Food items within each NSLP meal/food component (i.e., grain, meat/meat alternate, fruit, vegetable, and milk) were weighed on food scales (OXO Good Grips Stainless Steel Food Scale with Pullout Display, 11-pound) individually, prior to service, and recorded as initial weights. Participants were instructed to leave trays with remaining food on the table when finished. Researchers closely monitored the eating area during consumption. Upon exit of all participants, researchers collected trays and weighed each individual food item/meal component remaining. This weight was recorded and compared to the initial serving weight measured before service, which resulted in waste as a percent of initial serving. The plate waste of each meal subcomponent (i.e., grain, protein, fruit, vegetable, milk) was recorded and also averaged across all subcomponents to create a total average plate waste value. Two scales of the same brand were used before service and one scale was used for measuring food items after service to decrease instrumentation error. Photos of plate waste were also taken for additional verification of results, if needed.

Change in hunger from pre- to post-meal was used to determine level of satiety. Hunger was measured using the 5-point Likert scale [36]. This is a common scale used in mindful eating techniques, eating disorder nutrition therapy, and diabetes nutrition therapy. The scale was developed by Kaiser Permanente Santa Clara [36]. Participants arrived at scheduled meal sessions having fasted (no food or calorie-containing beverages) for two hours and to be consistent in their morning routine for each meal session. The hunger scale was completed using a single question (“How hungry are you?”} asked by trained researchers twice at each of the three meal sessions, first before leaving the food service area and consuming the meal, and a second time after consuming the meal, but before leaving the testing area. Responses were coded for analysis (i.e., stuffed = 1, full = 2, comfortable = 3, hungry = 4, ravenous = 5). Change in hunger was determined by subtracting pre-meal hunger from post-meal hunger.

#### 2.4.2. Feasibility

Meal cost was determined by first dividing the cost of a full package of a food item or ingredient from grocery store receipts, by the number of servings in that package, to determine the cost of one serving of each ingredient or food item purchased. The cost of one serving of each ingredient was then multiplied by the number of servings of that ingredient used to prepare each recipe, to determine the cost of the ingredient in the recipe. The cost of each ingredient in each recipe was totaled to obtain a recipe cost, which was then divided by the number of portions prepared by that recipe. With the cost of each food item and each recipe portion determined, these were totaled for each food item and recipe portion making up a meal, to determine the meal cost.

Preparation time was determined using the start and end time of each step of the preparation process of a food item or a recipe. Meals were prepared by undergraduate and graduate student research staff. The time to perform each preparation step was totaled for each food item or recipe, to determine the total time to prepare each food item or recipe, which was then totaled for each meal, resulting in the final meal preparation time.

Skill and equipment needed to prepare meals were determined by an experienced school foodservice director and Registered Dietitian (KH), based on experience with job descriptions and duties of staff and with equipment for large-scale cooking in a school foodservice environment. The researcher evaluated each recipe and food item within a meal to determine the skills and the types of equipment, small and large, required to prepare each meal.

### 2.5. Statistical Analysis

Statistical analyses were performed using SPSS analytic software (version 25, IBM Corporation, Armonk, NY, USA). Descriptive statistics included means and standard deviations, and proportions for baseline characteristics and acceptability and feasibility measures. One-way ANOVA and chi-squared test were used to determine differences in baseline characteristics between groups. Presence of an order effect was investigated using one-way ANOVA for differences in acceptability (overall taste test survey scores, total average plate waste percentage, change in hunger) between groups. Cronbach’s alpha, with a cut-point of 0.6, was utilized to ensure that taste test survey and plate waste assessment subcomponents were consistently measuring the same construct. Milk percent plate waste was the only item excluded in general and also from total average plate waste percentage, as it had a Cronbach’s alpha < 0.6, and was not consistent with the other measures of plate waste. For acceptability comparisons, one-way ANOVA was used to determine significant differences in total taste test score, total average plate waste percentage, and change in hunger between overall BPSL and overall TSL and also between BPSL in meal condition 1, as compared to meal condition 3. Analyses were repeated using ANCOVA to adjust for possible confounders, including sex, grade level, BMI percentile, and group. Binary logistic regression, with entry method, was utilized to determine whether any participant characteristics predicted selection of the BPSL in meal condition 3. Characteristics in the regression analysis included sex (two groups: male or female), grade level (three groups: K + 1st, 2nd + 3rd, 4th + 5th), BMI percentile (three groups: healthy weight < 85th percentile, overweight 85–95th percentiles, obese > 95th percentile), fruit consumption (two groups: < 1 serving/day, ≥1 serving/day), vegetable consumption (three groups: <0.5 servings/day, 0.5–1 serving/day, >1 serving/day), and added sugar consumption (three groups: 0–8 g/day, >8–16 g/day, >16–27 g/day). For feasibility comparison, one-way ANOVA was used to determine differences in preparation time and cost of meals between overall BPSL and overall TSL. Follow-up analyses were performed to determine whether there were any significant differences in taste test survey subcomponents (taste, smell, appearance, and service recommendation) and meal component plate waste assessment (fruit, vegetable, grain, protein, and milk). Level of significance was set at 0.05, with Bonferroni correction used for multiple comparisons. Parametric assumptions were checked for normality and equality of variance using Levene’s test and Browne-Forsythe test. Variance inflation factors, with cut-point < 5, were checked for all variables before performing regression analysis.

## 3. Results

### 3.1. Participant Characteristics

Forty-three participants expressed interest in the current study, with thirty-six (84%) completing all three meal sessions. During screening, five participants were excluded due to food allergy, inability to make scheduled initial assessment appointment times, and unwillingness to undergo a physical assessment. Of the 38 remaining interested participants, two started, but did not complete the study (5% dropout rate). Dropouts were due to new diagnosis of food intolerance and scheduling communication issues. Thirty-six participants completed the study and were included in this analysis. Twenty-four participants attended all three meal sessions as scheduled, while twelve participants attended at least one make-up session.

Participant characteristics of those completing the study can be found in Table 2. There were no significant between-group differences for baseline characteristics including gender, age, grade level, or ethnicity. There was a significant difference between groups for weight (*p* = 0.003), where group 2 was heavier than groups 1 and 3. There were no other significant anthropometric differences between groups.

### 3.2. Differences in Acceptability between BPSL and TSL

#### 3.2.1. Taste Test Evaluation

**Figure 3:**
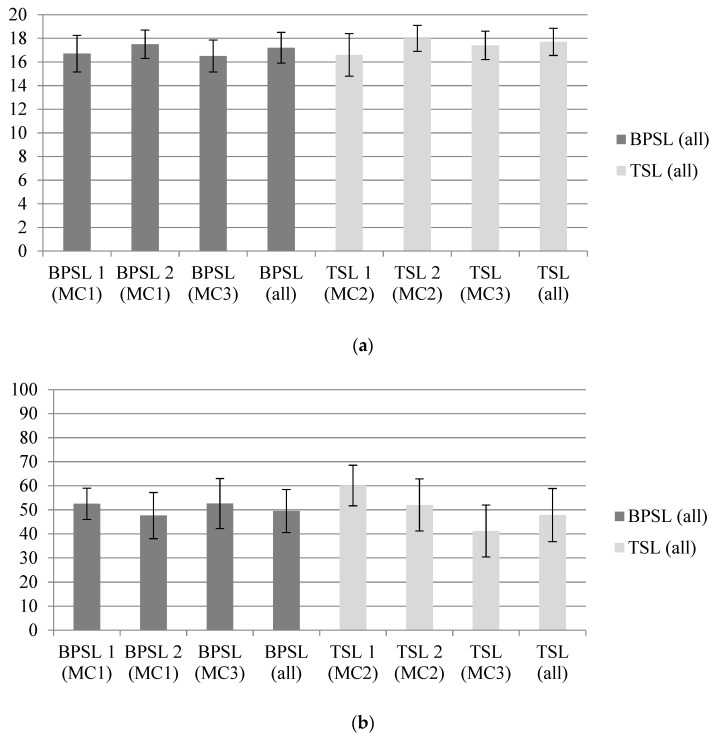

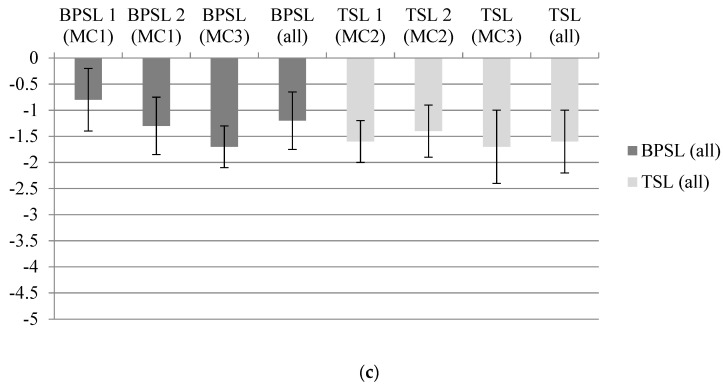
Comparison of acceptability by meal type: (**a**) total taste test score; (**b**) average total plate waste; (**c**) change in hunger. There were no significant differences between meal types for total taste test score, average total plate waste, and changes in hunger after adjusting for sex, grade level, BMI percentile, and group (*ps* > 0.017). MC = meal condition. Max scores: total taste test score = 20 points, average total plate waste = 100%, change in hunger = 5 points from pre- to post-meal consumption. Error bars = standard deviation.

Taste test results are summarized in Table S1 and can be visualized in Figure 3. There were no significant differences in total taste test score (*p* = 0.420) between overall BPSL and overall TSL before controlling for confounders. Following adjustment for sex, grade level, BMI percentile, and group, total taste test score differences remained non-significant (*p* = 0.226). Post-hoc analysis of individual taste test scoring subcomponents revealed no significant differences between overall BPSL and overall TSL for taste, smell, appearance, or service recommendations, before or after adjusting for covariates (*ps* > 0.013).

#### 3.2.2. Plate Waste Assessment

Plate waste results are summarized in Table S2 and can be visualized in Figure 3. There was no significant difference in average total plate waste (*p* = 0.582), before controlling for confounders. There were also no significant differences after adjusting for sex, grade level, and group individually or all covariates collectively. When controlling for BMI percentile alone, there was a significant difference in average total plate waste, such that the obese participants wasted the least of BPSL, followed by the healthy weight participants and then the overweight participants (adjusted mean = 50.4 ± 2.0%, *p* = 0.006). Post-hoc analysis of individual meal component waste revealed no significant differences between overall BPSL and overall TSL in fruit, vegetable, grain, protein, or milk waste, before or after adjusting for covariates (*ps* > 0.01).

#### 3.2.3. Changes in Hunger

Changes in hunger can be visualized in Figure 3. There was no significant difference in change in hunger between overall BPSL and overall TSL (*p* = 0.197) before controlling for confounders. There was also no significant difference after adjusting for sex, grade level, BMI percentile, and group individually and collectively (*ps* > 0.05).

#### 3.2.4. Differences by Group

There was a significant difference in acceptability by group, suggesting an order effect. Total taste test score was significantly different, such that Group 2, which completed the two meal conditions including BPSL first before completing the meal condition with only TSL, had higher total taste test scores for BPSL than Groups 1 and 3 (*p* = 0.003, Group 1: 16.5 ± 2.0, Group 2: 18.7 ± 1.9, Group 3: 18.0 ± 2.2). Change in hunger (*p* = 0.647) and average total plate waste (*p* = 0.034) were not significantly different between groups. Because these results indicate a possible order effect, group was included as a covariate in subsequent analyses.

### 3.3. Influence of Presence of Competitive Foods on Acceptability

Aim three was to investigate whether offering BPSL alongside less healthful, competitive foods (TSL) influenced acceptability of the BPSL. To investigate this matter, results from meal selection, taste test, plate waste, and hunger scale for BPSL served in meal condition 1 with only BPSL meals served (BPSL1 and BPSL2) were compared to results for BPSL served in meal condition 3 alongside TSL. These data are presented in Tables S1 and S2, and Figure 3.

#### 3.3.1. Meal Selection

Meal selection for meal condition 3 was also investigated. There was a significant difference in meal type selection in meal condition 3. The TSL meal option was selected significantly more often than the BPSL meal option (TSL = 83.3%, BPSL = 16.7%, *p* = 0.001).

Regression analysis was performed to determine whether any participant characteristics predicted selection of BPSL over TSL in meal conditions 3. Sex, grade level, BMI percentile, fruit consumption, vegetable consumption, and added sugar consumption were included in models. Neither model, forcing all variables together, or step-wise with dietary factors first, followed by participant characteristics, showed significant predictors for selecting BPSL over TSL in meal condition 3 (model with all variables at once: sex *p* = 0.781, grade level *p* = 0.460, BMI percentile *p* = 0.979, fruit consumption *p* = 0.152, vegetable consumption *p* = 0.441, and added sugar consumption *p* = 0.300). Table 3 presents a regression table with odds of participants selecting the BPSL in meal condition 3 by baseline characteristic. Participants were not more likely to choose BPSL in meal condition 3 by any of the investigated characteristics.

#### 3.3.2. Taste Test Evaluation

There were no significant differences in total taste test scores when comparing BPSL served in meal condition 1 and BPSL served in meal condition 3 (*ps* > 0.017), before or after controlling for all confounders collectively. When controlling for BMI percentile alone, there was a significant difference in total taste test score, due to overweight participants not selecting the BPSL over the TSL in meal condition 3 (adjusted mean = 17.1 ± 0.6, *p* = 0.015). Post-hoc analysis of individual taste test scoring components revealed no significant difference in individual taste, smell, appearance, or service recommendation before or after controlling for all confounders collectively. Again, when controlling post-hoc analysis for BMI percentile alone, there was a significant difference in survey score for smell (*p* = 0.005).

#### 3.3.3. Plate Waste Assessment

There was no significant difference in average total plate waste (*p* = 0.760), before or after controlling for confounders, between BPSL in meal condition 1 and BPSL in meal condition 3. Post-hoc analysis of individual meal component plate waste revealed no significant difference in fruit, vegetable, grain, protein, or milk waste before or after controlling for all confounders collectively. When controlling post-hoc analysis for BMI percentile alone, there was a significant difference in protein waste (*p* = 0.001).

#### 3.3.4. Change in Hunger

There were no significant differences in change in hunger between BPSL in meal condition 1 and BPSL in meal condition 3 (*p* = 0.308) before controlling for confounders. There were also no significant differences after adjusting for sex, grade level, BMI percentile, and group individually and collectively.

### 3.4. Qualitative Results Regarding Acceptability

Several questions were asked in an open-answer format to gather qualitative data regarding acceptability of meals. A record of all comments and responses can be found in Tables S3–S8. The first question was upon selection of the meal regarding what reason the participant had for choosing that meal and what reason they had for not choosing the other meal option. The majority of responses, found in Tables S3–S5, point to favoring or disliking one specific food item. Some food preferences also became apparent. The participants highly favored hot dogs and pizza over most other foods, while disliking both forms of broccoli provided. The rest of the likes and dislikes as rationale for selection were widely varied.

The second question was whether the participants had any comments at the end of their taste test survey. Recorded comments can be found in Tables S6–S8. There were no common themes to the comments recorded. Comments were mainly children practicing use of sensory descriptors for foods (i.e., bread is soft, chicken is crunchy) and stating likes or dislikes, which were documented quantitatively in selection, consumption, and taste test score data.

### 3.5. Differences in Feasibility between BPSL and TSL

#### 3.5.1. Meal Cost

Cost data are presented in Table 4. Overall average BPSL cost was $0.12 more per meal than TSL, which is a 3% difference, however, this was not a significant difference (*p* = 0.783).

#### 3.5.2. Preparation Time

Preparation time data can also be found in Table 4. BPSL, overall, required significantly longer preparation time than TSL (BPSL: 267 min, TSL: 60 min, *p* = 0.026).

#### 3.5.3. Skill

A breakdown of skills needed to prepare each meal and meal item can be found in Appendix A. There were two skills found to be common to both BPSL and TSL. There were four additional skills needed by school foodservice staff to prepare BPSL—follow and scale standardized recipes, which requires basic math skills; weigh and measure recipe ingredients accurately, which requires basic math skills and knowledge of common weights and measures; follow food safety procedures for the handling of fresh ready-to-eat (RTE) produce; possess knowledge of baking techniques. These additional skills may not greatly increase training needs or skill level of applicants/employees.

#### 3.5.4. Equipment

A breakdown of large kitchen equipment and smaller kitchenware needed to prepare each meal and meal item can also be found in Appendix B. There were three pieces of larger kitchen equipment and four pieces of smaller kitchen equipment common to both BPSL and TSL preparation. BPSL required six additional pieces of larger equipment and four additional pieces of smaller equipment. These additional pieces of equipment are commonly found in most school foodservice environments and may not greatly increase equipment needs.

## 4. Discussion

The primary purpose of this study was to compare acceptability and feasibility of BPSL, of high DQ, to TSL, of moderate DQ. Overall, our results suggest that high DQ school lunches are acceptable to children in grades K–5, particularly when offered alongside a second high DQ meal choice. The results also suggest that high DQ school lunches are equally acceptable to elementary schoolchildren when served alongside a lower DQ meal choice, but will be selected much less often than the lower DQ option. Additionally, there was evidence suggesting that weight status may impact acceptability of high DQ school lunches, however, when adjusting for other potential confounders in addition to weight status, this difference was no longer significant. Additionally, there was a significant order effect, such that the group of participants who completed the two meal conditions including BPSL first, followed by the TSL only condition, had higher total taste test scores for BPSL than those of groups exposed to TSL earlier in the meal condition order. These results suggest that being exposed to higher DQ school lunches before less healthful, competitive foods may improve acceptability of the higher DQ options.

The third aim of the current study, related to acceptability, was to investigate whether the presence of both meal types in one meal setting (choice) influenced the acceptability of the best practice school lunches. The results suggest that high DQ school lunches are equally acceptable to elementary schoolchildren when served alongside a lower DQ meal choice, in terms of taste preference and plate waste, but will be selected much less often than the lower DQ option. Again, there was evidence that weight status may impact acceptability of high DQ school lunches when served alongside competitive foods of lower DQ. These differences were also no longer significant after adjusting for other potential confounders in addition to weight status. To supplement this quantitative data on acceptability, results are corroborated and even explained by selection and taste test survey comments. Based on these comments, the participants did not appear to notice any difference, or favor any particular meal type when served separately. However, when BPSL and TSL were served simultaneously, a useful theme became apparent that less healthful, competitive foods would consistently be chosen over more healthful options, but that more healthful options were very appealing and would have happily been chosen had the less healthful, competitive option not been present.

In terms of feasibility, this study adds to the current body of research on cost comparisons, and to our knowledge, is the first to compare time, skill and equipment needed to prepare high DQ school lunches. With a 3% higher meal cost, the BPSL was not significantly more expensive than the TSL, contrary to common perceptions. In contrast, commonly reported time requirements for high DQ lunches were confirmed by the current study, as BPSL took significantly longer to prepare as compared to TSL. Caution should be taken, however, when interpreting this difference for several reasons. The total times calculated include every minute of preparation, whether multiple tasks were done simultaneously (multi-tasking) or not. Also, food was prepared by undergraduate and graduate students, who are not experienced cooks. Additionally, food was prepared using small, dated, home-style kitchens with no use of large-scale cooking equipment or space (i.e., Robot Coupe, double ovens, steam kettles, etc.). It should also be noted that ample, and even excess time was set aside to prepare each meal, and thus efficiency was not a priority, instead focusing on accuracy. Finally, recipes chosen for the BPSL were minimally processed and utilized very few value-added, convenience products. Value-added product purchase (i.e., pre-chopped fresh broccoli) and use of large-scale cooking equipment (i.e., Robot Coupe) would reduce time required to prepare BPSL meals. Thus, the time difference presented here may not be ecologically valid in comparison to that which a school foodservice operation would actually experience. There were a few additional skills and large and small pieces of kitchen equipment needed to prepare BPSL as compared to TSL, however the additional equipment needs are for equipment commonly found in school foodservice operations and would likely not require major acquisitions of additional equipment. Similarly, additional skills needed to prepare BPSL would not be such that they greatly impact training or hiring practices. Caution should be taken when interpreting feasibility results, as scaling these results from a small-scale, lab-style operation to a large-scale, multi-unit school foodservice operation is unlikely to be clear and linear.

To our knowledge, this is one of the first studies to extensively investigate differences in the acceptability and feasibility of best practice as compared to typical school lunches. Plate waste percentages were similar in the current study to those of previous studies by Marlette et al. (2005) [20], Smith and Cunningham-Sabo (2013) [19], Cohen et al. (2014) [21], Byker et al. (2014) [22], and Gase et al. (2014) [24]. Marlette et al. (2005) investigated the influence of food preparation methods and competitive foods on school lunch plate waste of sixth graders in three Kentucky middle schools. Results of this study showed that competitive food purchases significantly affected plate waste of fruit, grain, meat, and mixed dishes and that plate waste was highest for those purchasing competitive foods. Additionally, results showed the impact of competitive food purchases was the greatest on waste of fruits and vegetables [20]. Similarly, Smith and Cunningham-Sabo (2013) investigated impact of the offer service style, where students can refuse some reimbursable meal components, and saw greater waste of higher DQ fruits and vegetables than those of lower DQ [19]. Byker et al. (2014) and Gase et al. (2014) measured what meal components and foods students wasted in general, within an actual lunchroom setting with reimbursable and competitive foods available, and found higher fruit and vegetable component waste [22,24]. An additional study looking at impact of competitive foods by Cluss et al. (2014) found that children consumed more healthful food items in the lunchroom when less healthful options were removed [26]. Collectively, the current results, corroborate previous results, suggesting that competitive foods impact healthier food acceptability. In the same children, under different conditions in the current study, the BPSL was acceptable, yet only 17% selected the BPSL when TSL was also offered. However, in the current study, there were no other acceptability differences between healthier lunches and less healthful, competitive lunches. This could be due to the smaller amount of options available, serve style of meals as compared to offer, and age groups investigated. Additionally, there were no significant differences in plate waste, before or after adjusting for all covariates, between BPSL and TSL in the current study. These results also support results of a study by Cohen et al. (2014) investigating differences in selection and consumption of meal components following implementation of the new NSLP nutrition standards [21]. Cohen and colleagues found increased consumption of vegetables and no other significant differences in meal component consumption with school lunches meeting the new 2012 NSLP nutrition standards as compared to meals meeting previous standards. Thus, no significant increase in waste was seen with higher DQ school lunches [21]. The current study additionally extended these results by investigating not just plate waste, but also taste test preference and change in hunger, with higher DQ school lunches.

The results of the current study also support and extend the existing body of literature on the feasibility of higher DQ school lunches. A study by Trevino et al. (2012) investigated the impact of improving the DQ of school lunches in a 3-year randomized cluster, primary prevention trial, in 42 middle schools over five states, on revenues and expenses [27]. Authors reported that there was no significant difference in revenues or expenses, and that there was a trend for intervention schools with higher DQ lunches, to have higher excess revenue over expense ($3.5 million) than control schools ($2.5 million) over the 3-year intervention [27]. A study by Cohen et al. (2016) looked at a sample from the NOURISH study to examine changes in school food revenue and participation rates with implementation of school lunch guidelines that were stricter than the NSLP (i.e., decrease in less healthful, competitive food options available and overall higher DQ school lunches) [25]. Results indicated that there was an initial small loss of overall revenue in year one due to loss of revenue from competitive foods, but overall revenue returned to baseline year two due to increase in school meal revenue. There was no decline in participation rates [25]. These results are supported by the current study indicating that there are no statistically significant cost differences between meal types in addition to no significant overall differences in the acceptability of meal types. A study by Cluss et al. (2014) investigating the impact of offering healthier foods in the lunchroom, found that food costs increased by about 15% and that participation decreased by 5–6% over five years during the intervention [26]. The current study challenges these reported cost differences, although the current study is a short-term analysis, whereas the Cluss et al. (2014) study was a long-term analysis. This difference in results could be due to numerous factors within the school food environment studied, including quality of food, presentation style, characteristics of the student population, and perceptions of school foodservice and teaching staff, to name a few.

### 4.1. Strengths

There were several strengths of the current study. The randomized crossover trial study design allowed for control for a potential order effect on acceptability. The study design also allowed us to determine the impact of choice on the acceptability of the high DQ school lunch options, which provides important context for determining acceptability in an offer setting and in a lunchroom with competitive foods available. A variety of measures were utilized for determining acceptability and feasibility. There was a conscious effort to eliminate bias and to ensure a lack of behavioral techniques in service of lunches that could impact selection, preference, or consumption. All researchers were trained by the principal investigator in survey methods and on appropriate professionalism during interactions with participants, specifically not to influence choices or responses of participants while assisting with meal service and completing surveys. Actions were also taken to ensure that meal presentation style was consistent between all meals offered and between meal sessions, so that presentation was not a confounding factor. Performing the trial in a lab setting allowed for isolation of meal type in impacting differences investigated, similar to an efficacy trial. Despite being in a lab setting, attempts were taken to create an environment similar to a lunchroom and to create meals similar to lunches served within a school lunchroom. The current study also included the youngest and broadest age group, elementary school-aged children, which is the earliest age group with which interventions can occur for the biggest prevention impact. Finally, a former foodservice director and Registered Dietitians were investigators on the current study, with knowledge on the NSLP nutrition standards and school foodservice operations.

### 4.2. Limitations

As with any study, there were limitations that require caution when interpreting the current study findings. A convenience sample was used to obtain participants, which may limit generalizability beyond our study sample. Sampling occurred mainly in the Manhattan, KS, area, which is a generally higher socioeconomic status area in Kansas and which may have been exacerbated by the inclusion of several participants who were children of Kansas State University faculty members. Higher household income and higher education level of parents and participants could bias results to having higher acceptability of BPSL. Additionally, the majority of participants were Caucasian, which again limits generalizability of results. The study was performed with elementary school-aged children, and thus results are not generalizable to middle or high school age groups. Further, the service style for meals was serve, and not offer. The serve style was most appropriate for initial investigations for the purposes of the current study, to isolate independent variables. The offer service system of meals could result in different acceptability of meals, as participants would have the ability to select different options for each meal component and to refuse up to two components as compared to selecting from two complete meals. Based on selection rationales and taste testing survey comments, it appears that some individual food items may have impacted selection of entire meals. Thus, repeating the study with the offer service system may impact meal item selection. Performing the current study in a lab could be considered a strength methodologically for isolating effects of independent variables, however it could also be considered a limitation as the lab setting is not ecologically valid for school foodservice operations. Thus, caution must be taken in generalizing results to actual school foodservice settings. Additionally, post-hoc power analysis was completed as no truly comparable studies could be found to accurately determine an adequate sample size for all analyses. The post-hoc power analysis showed that the study was under-powered and thus may not have been able to detect all significant differences between meal types or conditions.

### 4.3. Directions for Future Research

There are several directions for future research based on the current study. Future research should examine actual taste preference and sensory aspects of meals for acceptability, not just plate waste. It would also be of benefit to investigate the differences in acceptability, especially selection differences, when a larger variety of lower DQ options are available, not including favorites like hot dogs and pizza. This could further elucidate the selection between BPSL and TSL conditions. Future studies should also perform further analysis of skill and equipment needs and assess the cost implications of such differences. Time needed to prepare varying DQ meals should also be further investigated within the school foodservice setting. Additional validated measures for preference and feasibility of school lunches are needed. Future research should also investigate implications of the offer system, as compared to serve style used in the current study, on the acceptability of higher DQ school lunches.

## 5. Conclusions

These results indicate that there are no differences in the acceptability, and minimal differences in the feasibility (except time) of high DQ school lunches as compared with less healthful, competitive options. Thus, perceived concerns/barriers related to lower acceptability and higher cost of improved DQ school lunches, might not be actual barriers. Time differences, however, were significant and may be a barrier to improving DQ of school lunches. An important finding from the current study was that when higher DQ and less healthful, competitive foods are served concurrently, the less healthful, competitive foods, may be selected more often than the higher DQ options. Additionally, earlier exposure to higher DQ lunches, before less healthful, competitive options, may improve their acceptability. These results may inform key school lunch stakeholders including policy-makers seeking to further improve DQ provided by NSLP nutrition standards, and to combat the recent relaxation of NSLP nutrition standards. Given that the overall goal of the NSLP is to provide healthy food to children, these results suggest that further investigation of whether schools should serve competitive foods in the lunchroom is needed.

## Figures and Tables

**Table 1:** Meals served for each meal condition.

NSLP Meal Component	Meal Condition 1		Meal Condition 2		Meal Condition 3	
	BPSL 1	BPSL 2	TSL 1	TSL 2	BPSL	TSL
Meat/Meat Alternate	Oven fried chicken *	Homemade cheese pizza *	Frozen chicken nuggets	Frozen cheese pizza	BBQ pulled pork *	Beef hot dog
Grain	Whole grain cornbread *	Whole grain crust *	White roll	(crust)	Whole grain slider buns	White hot dog bun
Vegetable	Broccoli salad *	Mixed greens salad with carrots, tomato, cucumber	Broccoli with cheese sauce	Carrots with ranch dip	Asian coleslaw *	Frozen French fries
Fruit	Grapes	Clementine	Pineapple fruit cup in 100% fruit juice	Mandarin orange fruit cup in 100% fruit juice	Apple slices	Peach fruit cup in 100% fruit juice
Milk	1% low fat milk, plain	1% low fat milk, plain	1% low fat milk, plain	1% low fat milk, plain	1% low fat milk, plain	1% low fat milk, plain

* Made from scratch. Recipes available upon request.

**Table 2:** Participant characteristics, all participants and by group.

Characteristic		All Participants	Group 1 (*n* = 11)	Group 2 (*n* = 11)	Group 3 (*n* = 14)
**Proportion (%)**					
Grade Level	Kindergarten	16.7%	18.1%	28.6%	16.2%
	1st	16.7%	18.1%	21.4%	16.2%
	2nd	16.7%	9.1%	21.4%	16.2%
	3rd	33.3%	36.4%	7.1%	24.3%
	4th	8.3%	9.1%	7.1%	16.2%
	5th	8.3%	9.1%	14.3%	10.8%
Sex	Female	50%	54.5%	50%	59.5%
	Male	50%	45.5%	50%	40.5%
Ethnicity	Caucasian	83.3%	81.8%	85.7%	78.4%
	Hispanic	0	0	14.3%	2.7%
	African American	0	0	0	5.4%
	Native American	0	0	0	0
	Asian/Pacific Islander	8.3%	9.1%	0	5.4%
	Other	8.3%	9.1%	0	8.1%
BMI Percentile Category	<85th, Healthy	69.4%	81.8%	45.5%	78.6%
	85–95th, Overweight	13.9%	18.2%	18.2%	7.1%
	>95th, Obese	16.7%	0	36.4%	14.3%
Mean ± Standard Deviation					
Age (years)		7.7 ± 1.7	7.5 ± 1.7	8.3 ± 1.1	7.3 ± 2.1
Height (cm)		130.2 ± 10.5	127.1 ± 10.4	136.7 ± 5.8	127.6 ± 11.7
Weight (kg)		30.2 ± 8.3	26.1 ± 6.2	36.7 ± 7.6 *	28.2 ± 7.6
BMI Percentile		61.4 ± 30.2	43.4 ± 32.6	77.5 ± 22.0	63.0 ± 27.7
Waist Circumference (cm)		56.1 ± 11.7	49.3 ± 16.5	61.6 ± 8.5	57.0 ± 6.2

* Indicates a statistically significant difference between groups (*p* < 0.004).

**Table 3:** Odds of selecting BPSL as opposed to TSL in meal condition 3 by baseline characteristic.

Baseline Characteristic	Odds Ratio ^+^ (95% Confidence Interval)
Sex	
Male *	1.00
Female	0.58 (0.10–3.38)
Grade Level	
K & 1st *	1.00
2nd & 3rd	0.50 (0.07–3.65)
4th & 5th	0.33 (0.03–3.84)
BMI Percentile	
Healthy (<85th) *	1.00
Overweight (85–95th)	0.34 (0.02–7.11)
Obese (>95th)	0.80 (0.08–8.47)
Fruit Consumption	
Low (<1 serving/day) *	1.00
High (>1 servings/day)	0.21 (0.02–2.04)
Vegetable Consumption	
Low (<0.5 serving/day) *	1.00
Moderate (0.5–1 serving/day)	0.12 (0.01–2.60)
High (>1 serving/day)	1.25 (0.20–7.96)
Added Sugar Consumption	
Low (0–8 g/day) *	1.00
Moderate (8–16 g/day)	0.14 (0.01–3.28)
High (>16 g/day)	2.26 (0.32–15.76)

* Reference category, ^+^ Unadjusted odds ratio.

**Table 4:** Comparison of preparation time and cost for meal types.

Meal	Food Items	Food Item Preparation Time (min)	Food Item Cost per Serving	Number of Servings Prepared	Total Meal Preparation Time (min)	Total Meal Cost per Serving
BPSL1 (MC1)	Chicken nuggets	177 (Start prep 11, marinade 105, finish prep 26, bake 35)	$0.50	18	379	$3.83
	Broccoli salad	120 (Prep 72, marinade 48)	$0.98			
	Cornbread	56 (Prep 18, preheat 14, bake 24)	$0.29			
	Grapes	26	$0.44			
	Milk	0	$1.62			
BPSL2 (MC1)	Pizza Crust	135 (Prep 43, set 92)	$0.10	18	239	$3.37
	Pizza Sauce	30 (Prep 15, cook 15)	$0.63 (sauce + cheese)			
	Pizza	61 (Prep 15, preheat 20, bake 26)	$0.73 (crust + sauce + cheese)			
	Salad	13	$0.84			
	Clementine	0	$0.18			
	Milk	0	$1.62			
BPSL (MC3)	Pulled pork	117 (Preheat 12, prep 10, bake 75, pull 20)	$1.21 (+BBQ sauce)	12	183	$4.20
	Slider buns	0	$0.18			
	Apples	9	$0.40			
	Coleslaw	57 (Prep 55, set 2)	$0.79			
	Milk	0	$1.62			
Overall BPSL					267	$3.80
TSL1 (MC2)	Roll (frozen)	28 (Preheat 13, bake 15)	$0.51 (+butter)	13	86	$4.36
	Broccoli (frozen) with cheese sauce (prepared)	12 (Prep 2, steam 9, mix with cheese sauce 1)	$0.57			
	Chicken nuggets (frozen)	46 (Preheat 14, bake 32)	$1.04 (+ketchup)			
	Fruit cup (canned)	0	$0.62			
	Milk	0	$1.62			
TSL2 (MC2)	Pizza (frozen)	37 (Preheat 17, bake 20)	$0.87	13	37	$3.39
	Carrots with dip	0	$0.28 (+ranch dip)			
	Fruit cup (canned)	0	$0.62			
	Milk	0	$1.62			
TSL (MC3)	Hot dog	31 (Boil water 21, cook 10)	$0.69	12	56	$3.28
	Hot dog bun	0	$0.45			
	French fries (frozen)	37 (Preheat 12, bake 25)	$0.21 (+ketchup)			
	Fruit cup (canned)	0	$0.31			
	Milk	0	$1.62			
Overall TSL					60	$3.68

## Supplementary Materials

The following are available online at https://www.mdpi.com/1660-4601/17/17/6299/s1, Figure S1: Sample taste test survey, Table S1: Summary and comparison of taste test acceptability of individual meals and meal types, Table S2: Summary and comparison of plate waste acceptability of individual meals and meal types, Table S3: Stated reasons for meal choices in meal condition 1 (2 BPSL), Table S4: Stated reasons for meal choices in meal condition 2 (2 TSL), Table S5: Stated reasons for meal choices in meal condition 3 (1 BPSL, 1 TSL), Table S6: Taste test survey comments for meal condition 1 (2 BPSL), Table S7: Taste test survey comments for meal condition 2 (2 TSL), Table S8: Taste test survey comments for meal condition 3 (1 BPSL, 1 TSL).

## Appendix A

Audit of skill needed to prepare meals.

**Meal Condition 1**

BPSL 1: Crispy baked whole grain chicken

- Knowledge of principles and methods of preparing food in large quantities according to appropriate food safety and sanitation procedures.
- Practice proper operation of equipment to assure safety and avoid damage to equipment and clean and sanitize equipment properly.
- Follow and scale standardized recipes; weigh and measure food ingredients accurately. Requires basic math skills and knowledge of common weights and measures.
- Operate the following kitchen equipment: oven, warmer, and cooler.
- Utilize the following kitchenware: knives, spatula, whisk, tongs, and measuring cups and spoons.

BPSL 1: Broccoli salad

- Knowledge of principles and methods of preparing food in large quantities according to appropriate food safety and sanitation procedures.
- Practice proper operation of equipment to assure safety and avoid damage to equipment and clean and sanitize equipment properly.
- Follow food safety procedures for the handling of fresh ready-to-eat (RTE) produce.
- Follow and scale standardized recipes; weigh and measure food ingredients accurately. Requires basic math skills and knowledge of common weights and measures.
- Utilize the following kitchenware: knives, spatulas, whisk, and measuring cups and spoons.

BPSL 1: Whole grain cornbread

- Knowledge of principles and methods of preparing food in large quantities according to appropriate food safety and sanitation procedures.
- Practice proper operation of equipment to assure safety and avoid damage to equipment and clean and sanitize equipment properly.
- Follow and scale standardized recipes; weigh and measure food ingredients accurately. Requires basic math skills and knowledge of common weights and measures.
- Operate the following kitchen equipment: electric mixers, and ovens.
- Utilize the following kitchenware: knives, spatula, whisk, and measuring cups and spoons.

BPSL 2: Whole grain pizza crust

- Knowledge of principles and methods of preparing food in large quantities according to appropriate food safety and sanitation procedures.
- Practice proper operation of equipment to assure safety and avoid damage to equipment and clean and sanitize equipment properly.
- Follow and scale standardized recipes; weigh and measure food ingredients accurately. Requires basic math skills and knowledge of common weights and measures.
- Knowledge of baking techniques.
- Operate the following kitchen equipment: electric mixer, dough hook, and oven.
- Utilize the following kitchenware: knives, pizza cutter, spatula, whisk, tong, rolling pin, and measuring cups and spoons.

BPSL 2: Homemade pizza sauce

- Knowledge of principles and methods of preparing food in large quantities according to appropriate food safety and sanitation procedures.
- Practice proper operation of equipment to assure safety and avoid damage to equipment and clean and sanitize equipment properly.
- Follow and scale standardized recipes; weigh and measure food ingredients accurately. Requires basic math skills and knowledge of common weights and measures.
- Operate the following kitchen equipment: stove top, tilt-skillets, and tilt-kettles.
- Utilize the following kitchenware: knives and measuring cups and spoons.

**Meal Condition 2**

TSL 1: Frozen chicken nuggets

- Knowledge of principles and methods of preparing food in large quantities according to appropriate food safety and sanitation procedures.
- Practice proper operation of equipment to assure safety and avoid damage to equipment and clean and sanitize equipment properly.
- Operate the following kitchen equipment: oven, warmer, and cooler.
- Utilize the following kitchenware: tongs.

TSL 2: Frozen pizza

- Knowledge of principles and methods of preparing food in large quantities according to appropriate food safety and sanitation procedures.
- Practice proper operation of equipment to assure safety and avoid damage to equipment and clean and sanitize equipment properly.
- Operate the following kitchen equipment: oven, warmer, and cooler.
- Utilize the following kitchenware: knives, pizza cutter, and spatula.

**Meal Condition 3**

BPSL: Pulled pork sliders

- Knowledge of principles and methods of preparing food in large quantities according to appropriate food safety and sanitation procedures.
- Practice proper operation of equipment to assure safety and avoid damage to equipment and clean and sanitize equipment properly.
- Follow and scale standardized recipes; weigh and measure food ingredients accurately. Requires basic math skills and knowledge of common weights and measures.
- Operate the following kitchen equipment: oven, warmer, and cooler.
- Utilize the following kitchenware: knives, tongs, and measuring cups and spoons.

BPSL: Asian cabbage salad

- Knowledge of principles and methods of preparing food in large quantities according to appropriate food safety and sanitation procedures.
- Practice proper operation of equipment to assure safety and avoid damage to equipment and clean and sanitize equipment properly.
- Follow food safety procedures for the handling of fresh ready-to-eat (RTE) vegetables and fruits.
- Follow and scale standardized recipes; weigh and measure food ingredients accurately. Requires basic math skills and knowledge of common weights and measures.
- Operate the following kitchen equipment: oven, food processor, and cooler
- Utilize the following kitchenware: knives, spatulas, whisks, and measuring cups and spoons

TSL: Hot dog on a bun

- Knowledge of principles and methods of preparing food in large quantities according to appropriate food safety and sanitation procedures.
- Practice proper operation of equipment to assure safety and avoid damage to equipment and clean and sanitize equipment properly.
- Operate the following kitchen equipment: oven, stovetop, warmer, and cooler.
- Utilize the following kitchenware: tongs.

TSL: Frozen French fries

- Knowledge of principles and methods of preparing food in large quantities according to appropriate food safety and sanitation procedures.
- Practice proper operation of equipment to assure safety and avoid damage to equipment and clean and sanitize equipment properly.
- Operate the following kitchen equipment: oven, warmer, and cooler.
- Utilize the following kitchenware: tongs.

## Appendix B

Audit of equipment needed to prepare meals.

**Meal Condition 1**

BPSL 1: Crispy baked whole grain chicken

- Kitchen equipment: oven, warmer, cooler
- Kitchenware: knives, spatula, whisk, tongs, measuring cups and spoons

BPSL 1: Broccoli salad

- Kitchenware: knives, spatulas, whisk, measuring cups and spoons

BPSL 1: Whole grain cornbread

- Kitchen equipment: electric mixers, ovens
- Kitchenware: knives, spatula, whisk, measuring cups and spoons

BPSL 2: Whole grain pizza crust

- Kitchen equipment: electric mixer, dough hook, oven
- Kitchenware: knives, pizza cutter, spatula, whisk, tong, rolling pin, measuring cups and spoons

BPSL 2: Homemade pizza sauce

- Kitchen equipment: stove top, tilt-skillets, tilt-kettles
- Kitchenware: measuring cups and spoons

**Meal Condition 2**

TSL 1: Frozen chicken nuggets

- Kitchen equipment: oven, warmer, cooler
- Kitchenware: tongs

TSL 2: Frozen pizza

- Kitchen equipment: oven, warmer, cooler
- Kitchenware: knives, pizza cutter, spatula

**Meal Condition 3**

BPSL: Pulled pork sliders

- Kitchen equipment: oven, warmer, cooler
- Kitchenware: knives, tongs, measuring cups and spoons

BPSL: Asian cabbage salad

- Kitchen equipment: oven, food processor, cooler
- Kitchenware: knives, spatulas, whisks, measuring cups and spoons

TSL: Hot dog on a Bun

- Kitchen equipment: oven, stove top, warmer, cooler
- Kitchenware: tongs

TSL: Frozen French fries

- Kitchen equipment: oven, warmer, cooler
- Kitchenware: tongs
