# Supplementary material for: Acceptability and Feasibility of Best Practice School Lunches by Elementary School-Aged Children in a Serve Setting: A Randomized Crossover Trial
Source: Int J Environ Res Public Health. 2020 Aug 29;17(17):6299. doi: 10.3390/ijerph17176299 (PMC7504630; doi:10.3390/ijerph17176299)
Supplement: Supplementary file 1 [file ijerph-17-06299-s001.pdf]

Figure S1. Sample taste test survey

Taste Test Survey

Color in the smiley face that best describes how you feel about the meal that you are tasting!

What do you think of the **color/look** of this meal?

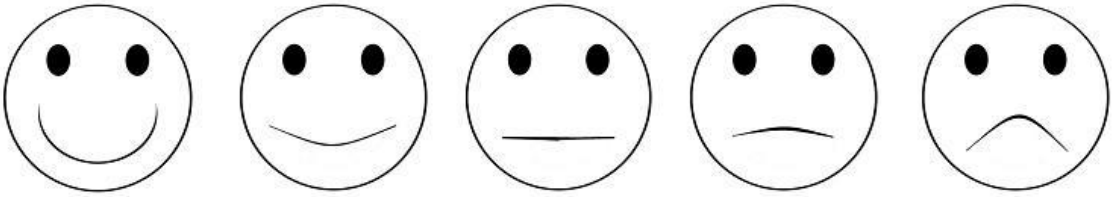

Very Good Good Just Okay Bad Very Bad

What do you think of the **taste/ flavor** of this meal?

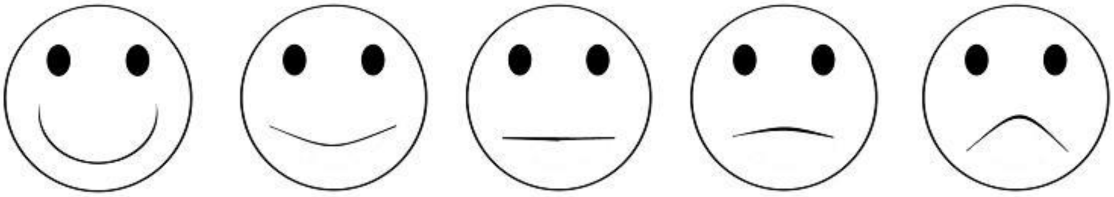

Very Good Good Just Okay Bad Very Bad

What do you think of the **smell** of this meal?

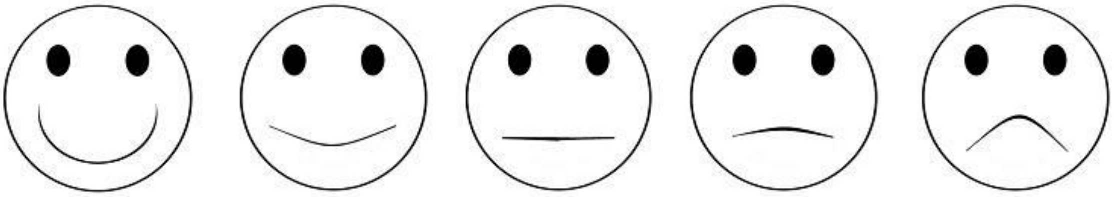

Very Good Good Just Okay Bad Very Bad

Do you think we **should serve this meal** on the lunch menu?

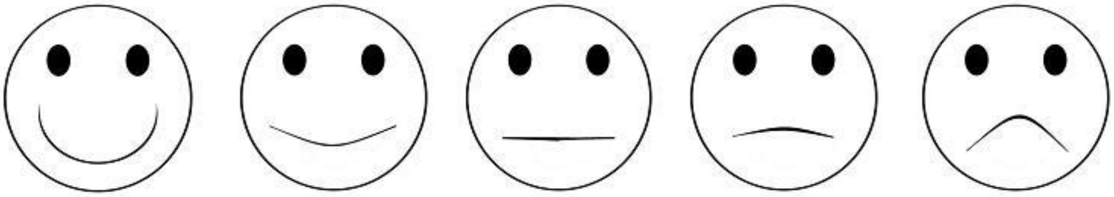

Very Good Good Just Okay Bad Very Bad

Any comments?

Thank you for tasting!

Table S1. Summary and comparison of taste test acceptability of individual meals and meal types

Meal	Taste Score^	Smell Score^	Appearance Score^	Serve at School Score^	Total Score~
			Mean ± Standard Deviation		
BPSL1 (MC1)	4.1±0.9	3.8±1.3	4.3±0.7	4.5±1.0	16.7±3.1
BPSL2 (MC1)	4.6±0.6	4.3±0.8	4.4±0.8	4.2±1.0	17.5±2.4
BPSL (MC3)	4.2±0.8	4.3±0.8	3.8±1.0	4.2±1.0	16.5±2.7
Overall BPSL	4.4±0.7	4.2±1.0	4.3±0.8	4.3±1.0	17.2±2.6
TSL1 (MC2)	4.7±0.5	3.7±1.1	4.1±0.9	4.0±1.4	16.6±3.6
TLS2 (MC2)	4.7±0.5	4.3±0.9	4.4±0.7	4.6±0.7	18.0±2.2
TSL (MC3)	4.4±0.7	4.3±0.9	4.3±0.8	4.4±0.8	17.4±2.4
Overall TSL	4.6±0.6	4.3±0.9	4.4±0.7	4.5±0.8	17.7±2.3

*MC = meal condition

+[pre-meal hunger score (out of 5)] – [post-meal hunger score (out of 5)]

^Maximum score of 5

~Maximum score of 20

**Significant difference (p<0.017 for total taste test score and average total plate waste, p<0.006 for taste test score subcomponents and meal component plate waste), after adjusting for sex, grade level, BMI percentile, and meal group

Table S2. Summary and comparison of plate waste acceptability of individual meals and meal types

Meal	Fruit Waste	Vegetable Waste	Grain Waste	Protein Waste	Milk Waste	Average Total Meal Waste
			%, Mean ± Standard Deviation			
BPSL1 (MC1)	4.7±11.7	81.8±27.3	49.1±43.6	54.3±33.3	72.7±33.7	52.5±13.0
BPSL2 (MC1)	47.2±40.1	72.9±28.4	28.4±33.1	28.4±33.1	60.9±44.9	47.6±19.2
BPSL (MC3)	61.6±40.7	76.2±26.4	27.0±36.8	26.7±37.5	71.6±39.1	52.6±20.8
Overall BPSL	39.1±40.2	75.5±27.5	33.1±36.5	34.3±34.8	65.3±41.2	49.5±17.9
TSL1 (MC2)	50.6±47.8	94.6±6.2	45.2±46.7	18.5±34.4	91.5±21.3	60.1±16.9
TLS2 (MC2)	51.7±38.1	70.7±29.4	34.7±40.2	34.7±40.2	68.2±40.7	52.0±21.7
TSL (MC3)	45.0±41.2	35.4±36.4	37.5±36.8	26.6±35.6	61.8±45.2	41.2±21.6
Overall TSL	48.5±40.3	56.8±37.9	36.4±38.7	28.3±36.8	68.8±41.3	47.8±22.1

*MC = meal condition

**Significant difference (p<0.017 for total taste test score and average total plate waste, p<0.006 for taste test score subcomponents and meal component plate waste), after adjusting for sex, grade level, BMI percentile, and meal group

Table S3. Stated reasons for meal choices in meal condition 1 (2 BPSL*)

Participant	Meal Chosen	Reason Meal Selected Was Chosen	Reason Meal Not Selected Was Not Chosen
001	BPSL1	Like the fruit and vegetable	Like the other one better
002	BPSL2	Pizza is my favorite	Don't like cornbread
003	BPSL2	Love pizza, just want pizza	Love cornbread, just want pizza
004	BPSL1	Like broccoli	Didn't want salad
005	BPSL2	Like oranges	Not feeling the nuggets today
006	BPSL1	Like cornbread and nuggets	Don't like salad and messy oranges
007	BPSL1	Like broccoli	Don't like cucumber
008	BPSL2	I really like pizza	Broccoli
010	BPSL2	Like pizza, lettuce, and cucumbers	Not a fan of broccoli
012	BPSL2	Love pizza and oranges	Don't like broccoli and raisins
014	BPSL2	It looks good	There's stuff in the broccoli
015	BPSL2	Like pizza	Don't like broccoli
016	BPSL2	Like pizza	No reason
017	BPSL2	Don't like broccoli	Don't like broccoli
018	BPSL2	No reason	More not like
019	BPSL2	Like salad	Don't like broccoli with ranch
020	BPSL2	Pizza and orange	No reason
021	BPSL2	The chicken looks different	Chicken nuggets looked strange
022	BPSL2	Like pizza	Don't like grapes
023	BPSL2	Really like pizza	Broccoli does not agree with me
024	BPSL2	Doesn't like dressing	There was dressing on the broccoli
025	BPSL1	Like cornbread	No reason
026	BPSL1	Like chicken and grapes	No reason
027	BPSL2	Really like pizza and cutie	Not like broccoli
028	BPSL2	Want pizza	Not like nuggets
029	BPSL1	Like bread	Not feeling pizza
030	BPSL2	Don't like other meal	Didn't like chicken and broccoli
031	BPSL1	Like nuggets, grapes, and cornbread	Don't like the vegetable
033	BPSL2	Like pizza	Don't like cornbread
034	BPSL2	Last time had nuggets	Last time had nuggets
035	BPSL2	Pizza, orange, and cucumber	Broccoli
036	BPSL1	Like cornbread and grapes	Don't like oranges
037	BPSL1	Like chicken	Like chicken
038	BPSL2	Like salad	No reason
040	BPSL2	Likes pizza more	Only like some chicken
041	BPSL2	Like most stuff	Don't know

*BPSL1 = Oven baked chicken nuggets, broccoli salad, grapes, whole grain cornbread, low-fat milk

BPSL2 = Whole grain cheese pizza, side salad with Italian dressing, clementine, low-fat milk

Table S4. Stated reasons for meal choices in meal condition 2 (2 TSL+)

Participant	Meal Chosen	Reason Meal Selected Was Chosen	Reason Meal Not Selected Was Not Chosen
001	TSL1	The pizza will make me too full	The pizza will make me too full
002	TSL2	Like pizza	Don't like pineapple
003	TSL2	Pizza is my favorite	Pizza is my favorite
004	TSL1	Like nuggets	Like pizza, just didn't want it
005	TSL2	Like oranges	Don't like pineapple
006	TSL2	Like pizza	Like pizza
007	TSL2	Not sure	Didn't want chicken
008	TSL2	Like pizza	Don't like broccoli
010	TSL2	Like pizza	Don't like cheesy broccoli or packaged pineapple
012	TSL2	Just really like pizza	Don't like cheesy broccoli and ketchup
013	TSL2	Like pizza	Don't like cheese on broccoli
014	TSL2	Like pizza	Like pizza
015	TSL2	Like pizza	Don't like chicken much
016	TSL2	Like pizza	Like pizza
017	TSL2	Like pizza	Don't like broccoli
018	TSL2	Looks better	The other one looks better
019	TSL2	Like pizza and ranch	Don't want a roll
020	TSL2	Like carrots and pizza	Like carrots and pizza
021	TSL2	Could smell pizza	Broccoli looks "eh"
022	TSL2	Like carrots	Don't like chicken
023	TSL2	Like the whole tray	Not comfortable with broccoli
024	TSL1	Hungry	Don't like ranch
025	TSL1	Like nuggets better	Like nuggets
026	TSL2	Like pizza, carrots, ranch, and oranges	Don't like broccoli
027	TSL2	Like pizza	Like pizza more
028	TSL2	Like pizza	No reason
029	TSL1	Not in the mood for pizza	Not in the mood for pizza
030	TSL2	Like pizza and fruit cup a lot	Don't like pineapples
031	TSL2	Like pizza	Like pizza
033	TSL2	Like oranges and carrots	No reason
034	TSL1	Like chicken	Don't know
035	TSL2	Like pizza	Don't like broccoli
036	TSL2	Like oranges	Don't like broccoli
037	TSL2	Want pizza	Want pizza
038	TSL1	Like this one	Had that one last time
040	TSL2	More vegetarian	Other one more vegetarian
041	TSL2	Like pizza and fruit	Like pizza and fruit

+TSL1 = Chicken nuggets, broccoli with cheese sauce, pineapple fruit cup, dinner roll, low-fat milk

TSL2 = Cheese pizza, carrots with ranch, mandarin oranges, low-fat milk

Table S5. Stated reasons for meal choices in meal condition 3 (1 BPSL, 1 TSL^)

Participant	Meal Chosen	Reason Meal Selected Was Chosen	Reason Meal Not Selected Was Not Chosen
001	TSL	Like fruit cup	Usually don't eat that much food
002	TSL	Like fries	Hate coleslaw
003	BPSL	Don't like hotdogs	Don't like hotdogs
004	TSL	Like fries	No reason
005	TSL	Like hotdogs, fries, and ketchup	Don't like pork
006	BPSL	Like pork and BBQ	Don't like messy fruit cup
007	TSL	Don't like salad much	Don't like salad much
008	TSL	Don't like coleslaw, love hotdogs	Don't like coleslaw
010	TSL	Like hotdog	No reason
012	TSL	Like hotdog	Don't like pork
014	TSL	Like French fries and peaches	Stuff in the bun looks weird
015	BPSL	Like food on that tray – salad, apples	Don't like peaches
016	TSL	Like hotdog	No reason
017	TSL	Don't want apples	Don't like apples
018	TSL	Don't like salad	Don't like salad
019	BPSL	Like the sandwiches	Don't like hotdogs
020	TSL	Like hotdog	Don't like it
021	TSL	Not much of a salad eater, like fries	Don't like salad
022	TSL	Like peaches	Like peaches
023	TSL	Like hotdogs, more used to hotdog than BBQ	Looks a little strange
024	TSL	Don't really like coleslaw	Too much food
025	TSL	Like hotdog, fries, and ketchup	Don't like BBQ pork
026	TSL	Really like hotdog and fruit cup	Don't like BBQ
027	TSL	Like hotdog and less healthy	Not into the salad
028	TSL	Only like the apples on the other tray	Like more on other tray
029	BPSL	Like coleslaw and apples	Don't like hotdogs
030	TSL	Like hotdog and fries	Other plate too tiny
031	TSL	Hotdogs are good	Don't like coleslaw or BBQ pork
033	TSL	I don't know	Don't like salad
034	TSL	Like hotdog and fries	Not into coleslaw
035	TSL	Like French fries	Don't like coleslaw
036	TSL	Like hotdogs	Don't like BBQ
037	BPSL	Like apples	Don't want it
038	TSL	Love hotdogs	If hotdogs weren't here, would eat the other meal
039	TSL	Like ketchup	Too much food
040	TSL	Like this one more but not sure about either	No reason
041	TSL	Like everything but fries	Like some but not all

^BPSL = BBQ pulled pork on whole grain slider buns, Asian coleslaw, apple slices, low-fat milk
TSL = Hot dog on whole grain-rich bun, French fries, peach fruit cup, low-fat milk

Table S6. Taste test survey comments for meal condition 1 (2 BPSL*)

Participant	Meal Chosen	Comments
001	BPSL1	"A little bit like the chicken nuggets but not as much"
002	BPSL2	"No carrots and purple stuff in salad."
003	BPSL2	"The pizzs is the Best"
004	BPSL1	none
005	BPSL2	"Nope ☺"
006	BPSL1	none
007	BPSL1	"Liked grapes. The chicken is crunchy. The bread is soft."
008	BPSL2	none
010	BPSL2	"Nope"
012	BPSL2	"AWSOME"
014	BPSL2	"pizza is good ip"
015	BPSL2	none
016	BPSL2	none
017	BPSL2	none
018	BPSL2	none
019	BPSL2	none
020	BPSL2	none
021	BPSL2	"The milk tastes like cheese. More cheese should be put on the pizza to prevent the sauce from going everywhere. Create a pepperoni option. I hate salad."
022	BPSL2	"The food is good every time I come I leave whith a happy Belly."
023	BPSL2	none
024	BPSL2	"Nothing frozen or not homemade"
025	BPSL1	none
026	BPSL1	none
027	BPSL2	none
028	BPSL2	none
029	BPSL1	"Realy good, awesome"
030	BPSL2	"Its good"
031	BPSL1	none
033	BPSL2	none
034	BPSL1	"Pleas put it in my schools menu because are food is DESCUSTING D':):"
035	BPSL2	none
036	BPSL1	none
037	BPSL1	none
038	BPSL2	"It was good probably people who like Italian ranch would like it...pizza taste kinda like calzone."
040	BPSL2	"I think on the salad it should have more dressing on it."
041	BPSL2	none

*BPSL1 = Oven baked chicken nuggets, broccoli salad, grapes, whole grain cornbread, low-fat milk
BPSL2 = Whole grain cheese pizza, side salad with Italian dressing, clementine, low-fat milk

Table S7. Taste test survey comments for meal condition 2 (2 TSL+)

Participant	Meal Chosen	Comments
001	TSL1	"I don't know why But I tasted something in the chicken nuggets that I did not really like."
002	TSL2	"The pizza was better than the circle pizza they serve at school"
003	TSL2	"It is amazing. It is very good."
004	TSL1	"NO BRocoi"
005	TSL2	"Nope"
006	TSL2	none
007	TSL2	"I no how to make pissa"
008	TSL2	none
010	TSL2	"NOPE"
012	TSL2	"This was AWSOME!"
013	TSL2	none
014	TSL2	"no."
015	TSL2	none
016	TSL2	none
017	TSL2	none
018	TSL2	"Petzza/all food at my school is taribel! I don't like red sas"
019	TSL2	none
020	TSL2	none
021	TSL2	"Serve this pizza vs the last pizza"
022	TSL2	"Thank's for the food"
023	TSL2	"The fruit bowl was a bit messy."
024	TSL1	none
025	TSL1	none
026	TSL2	none
027	TSL2	"I think it was Asome. PJ could we have pancakes next time."
028	TSL2	none
029	TSL1	"Awesome"
030	TSL2	none
031	TSL2	"I really really like that" (to the pizza) "I like everything"
033	TSL2	none
034	TSL1	"soggy Brocily."
035	TSL2	"nope"
036	TSL2	none
037	TSL2	none
038	TSL1	"No, not really."
040	TSL2	"I don't like ranch I like blue cheese I also dont like the oranges"
041	TSL2	none

+TSL1 = Chicken nuggets, broccoli with cheese sauce, pineapple fruit cup, dinner roll, low-fat milk

TSL2 = Cheese pizza, carrots with ranch, mandarin oranges, low-fat milk

Table S8. Taste test survey comments for meal condition 3 (1 BPSL, 1 TSL^)

Participant	Meal Chosen	Comments
001	TSL	none
002	TSL	"I would put the burgers with the fries."
003	BPSL	"the Berger are okay."
004	TSL	none
005	TSL	"nope. The ketchup is a little sour and the fires are so good!"
006	BPSL	none
007	TSL	none
008	TSL	"More sweets"
010	TSL	"No"
012	TSL	"AWSOME"
014	TSL	"My mom will plopobly wont be that mad at my choice because sometimes im allowed to have hotdogs"
015	BPSL	none
016	TSL	none
017	TSL	none
018	TSL	none
019	BPSL	"This was the best school lunch I have tasted."
020	TSL	none
021	TSL	"the fries are cold"
022	TSL	"The food was really good."
023	TSL	none
024	TSL	none
025	TSL	none
026	TSL	none
027	TSL	none
028	TSL	none
029	BPSL	none
030	TSL	none
031	TSL	none
033	TSL	none
034	TSL	"Peaches are little to sogy and slimy"
035	TSL	none
036	TSL	none
037	BPSL	none
038	TSL	"This meal was great I'm steel on the thought of very good I would love for this to be on the lunch menu at school. P.s. frys are a little cold"
039	TSL	"The peaches are sogy"
040	TSL	"I liked the sides but not the hotdog"
041	TSL	none

^BPSL = BBQ pulled pork on whole grain slider buns, Asian coleslaw, apple slices, low-fat milk

TSL = Hot dog on whole grain-rich bun, French fries, peach fruit cup, low-fat milk
